# Impact of rectal balloon‐filling materials on the dosimetry of prostate and organs at risk in photon beam therapy

**DOI:** 10.1120/jacmp.v14i1.3993

**Published:** 2013-01-07

**Authors:** Shiv P. Srivastava, Indra J. Das, Arvind Kumar, Peter A. S. Johnstone, Chee‐Wai Cheng

**Affiliations:** ^1^ Department of Health Sciences Purdue University West Lafayette IN; ^2^ Department of Radiation Oncology Reid Hospital & Health Care Services Richmond IN; ^3^ Department of Radiation Oncology Indiana University School of Medicine Indianapolis IN USA

**Keywords:** IMRT, rectal balloon, dosimetry

## Abstract

The use of rectal balloon in radiotherapy of prostate cancer is shown to be effective in reducing prostate motion and minimizing rectal volume, thus reducing rectal toxicity. Air‐filled rectal balloon has been used most commonly, but creates dose perturbation at the air‐tissue interface. In this study, we evaluate the effects of rectal balloon‐filling materials on the dose distribution to the target and organs at risk. The dosimetric impact of rectal balloon filling was studied in detail for a typical prostate patient, and the general effect of the balloon filling was investigated from a study of ten prostate patients covering a wide range of anterior–posterior and left–right separations, as well as rectal and bladder volumes. Hounsfield units (HU) of the rectal balloon filling was changed from −1000 HU to 1000 HU at an interval of 250 HU, and the corresponding changes in the relative electron density (RED) was calculated. For each of the HU of the rectal balloon filling, a seven‐field IMRT plan was generated with 6 MV and 15 MV photon beams, respectively. Dosimetric evaluation was performed with the AAA algorithm for inhomogeneity corrections. A detailed study of the rectal balloon filling shows that the GTV, PTV, rectal, and bladder mean dose decreased with increasing values of RED in the rectal balloon. There is significant underdosage in the target volume at the rectum–prostate interface with an air‐filled balloon as compared to that with a water‐filled balloon for both 6 MV and 15 MV beams. While the dosimetric effect of the rectal balloon filling is reduced when averaged over ten patients, generally an air‐filled balloon results in lower minimum dose and lower mean dose in the overlap region (and possibly the PTV) compared to those produced by water‐filled or contrast‐filled balloons. Dose inhomogeneity in the target volume is increased with an air‐filled rectal balloon. Thus a water‐filled or contrast‐filled rectal balloon is preferred to an air‐filled rectal balloon in EBRT of prostate treatment.

PACS numbers: 87.55.D‐, 87.55.de, 87.55.dk, 87.55.Gh, 87.55.kd

## I. INTRODUCTION

Adenocarcinoma of prostate is the most commonly diagnosed malignancy in men and is the second leading cause of cancer related mortality. Management of the prostate cancer may include mono or combination therapy, watchful waiting, radical prostatectomy, radiation therapy and androgen deprivation. Radiation therapy options include brachytherapy and external beam radiation therapy (EBRT). In EBRT, intensity‐modulated radiation therapy (IMRT) has become the standard of care as it allows dose optimization to spare the surrounding normal tissues while delivering a full dose to the target volume. However, the accuracy of radiation therapy treatments of prostate cancer is challenging due to the uncertainties involved with patient setup and organ motion. In prostate IMRT, the varying state of rectal filling is a main factor in prostate gland motion and contributes to the majority of the organ motion.^(^ ^1^ ^–^ ^8^ ^)^ In addition to this interfractional motion, it has been reported that intrafractional motion of the prostate may also occur, and patients with large volume of rectal gas showed significant amount of rectal displacement (> 3 mm) in the superior–anterioposterior direction.^(^ ^9^ ^–^ ^15^ ^)^ However, organ motion can be minimized by using a rectal balloon and this is one of the reasons it is used routinely in particle beam therapy.

The use of rectal balloon has been found to be well‐tolerated and effective in reducing the intrafraction motion and improving the sparing of rectal wall by reducing the rectal volume in the high‐dose region, resulting in significant reduction in rectal toxicity.^(^ ^16^ ^–^ ^20^ ^)^ Air‐filled rectal balloons with volume varying from $40-100\;{\text{cm}}^{3}$ have been used most commonly for external beam prostate radiotherapy.^(^ ^21^ ^–^ ^25^ ^)^ Use of a water‐filled rectal balloon for prostate irradiation has been reported in proton therapy to reduce the rectal wall dose.^(^ ^26^ ^)^ While dosimetric effects of an air‐filled rectal balloon have been published in terms of dose‐volume studies on rectum and bladder, its effects on the coverage of prostate and PTV have not been sufficiently studied. An obvious drawback of an air‐filled balloon is the dose inhomogeneity created, especially at the air‐tissue interface, which results in a lack of charged particle equilibrium and a lack of scattered radiation. The aim of this study is to evaluate the effects of rectal balloon filling‐media on the dose distribution to GTV, PTV, rectum, bladder, and overlap of PTV to rectum.

## II. MATERIALS AND METHODS

At our hospital, prostate cancer patient is immobilized in a vac‐loc system and scanned on a GE CT simulator (GE Healthcare, Milwaukee, WI) with 2.5 mm slice spacing. The patient is instructed to maintain a full bladder for the CT scan and a rectal balloon is used to decrease the organ motion. Images are sent to the Eclipse treatment planning system (Varian Medical Systems, Palo Alto, CA) for planning. Target volumes and organs at risk are delineated in the TPS. The impact of rectal balloon filling on dose distribution was studied in detail for a typical prostate patient to give a critical evaluation of how change in the Hounsfield unit (HU) of the rectal balloon may affect the dosimetry in prostate planning. A generalization of the effects of rectal balloon filling on dose distribution is then investigated by a similar dosimetric study for ten prostate patients covering the range of physical measures encountered in the clinic.

### A. Detailed study of effects of rectal balloon filling on dose distributions

The HU of the rectal balloon was changed from −1000 HU (air) through zero (water) to 1000 HU (dense contrast material) in intervals of 250 HU for each IMRT plan to simulate the various balloon fillings for patient #1 listed in Table 1. The corresponding change in the relative electron density (RED) for each assigned HU was determined from the tissue characterization curve obtained with an RMI phantom (Gammex Inc., Middleton, WI). One additional structure was defined at the interface of rectum and PTV, namely, an overlap volume (O) that is the intersection of PTV and rectum. Nine step‐and‐shoot IMRT plans with a 7‐field technique (RPO 334°, RPO 283°, RAO 231°, AP 180°, LAO 129°, LPO 77° and LPO 26°) were generated for both 6 MV and 15 MV photon beams for a Varian iX linear accelerator (old Varian‐Scale: 0° at 6 o'clock and 180° at 12 o'clock) with 0.5 cm Millennium multileaf collimator. The prescribed dose was 7920 cGy in 44 fractions to the 100% isodose line. The dose constraints consisted of minimum, maximum, and mean doses and specific dose‐volume criteria for each structure. In particular, V(PTV)95%≥95%, (95% of PTV receives at least 95% of prescribed dose). The desired dose‐volume constraints were kept the same for all nine plans for both energies. During the optimization process, a maximum of 300 iterations were allowed until a desired solution based on the dose‐volume constraints was achieved.

**Table 1:** Physical measures and volumes of interest of the ten selected patients.

	*AP Separation (cm)*	*Lateral Separation (cm)*	*Rectum Vol*. (${\text{cm}}^{3}$ *)*	*Bladder Vol*. (${\text{cm}}^{3}$ *)*	*Prostate Vol*. (${\text{cm}}^{3}$ *)*	*PTV Vol*. (${\text{cm}}^{3}$ *)*	*Overlap Vol*. (${\text{cm}}^{3}$ *)*
Pt1	20.4	37.3	145.4	305.6	100.1	220.4	7.4
Pt2	27.3	44.5	154.4	302.0	50.0	144.6	5.9
Pt3	21.2	36.1	165.1	179.8	144.4	299.2	5.3
Pt4	23.2	36.1	152.1	99.1	122.9	255.4	4.6
Pt5	19.6	38.3	144.9	64.9	57.7	145.4	4.2
Pt6	22.7	37.3	172.0	246.9	54.8	137.0	4.6
Pt7	23.4	38.2	156.0	128.6	81.1	151.4	4.9
Pt8	21.5	35.1	184.8	170.9	58.8	148.4	4.1
Pt9	23.5	40.3	147.4	200.7	61.3	153.5	2.6
Pt10	22.4	36.5	150.2	86.2	45.6	123.5	3.4
Average	22.5	38.0	157.2	178.5	77.7	177.9	4.7
STD	2.13	2.72	13.0	86.2	33.9	59.2	1.33

The overlap structure between PTV and rectum was not used in the IMRT optimization clinically, but was used in this study to track the dose‐volume variation for the different plans as a function of HU. The optimal fluence was converted into an “actual” fluence by using the leaf motion calculator, which designs the optimal leaf motion patterns. The dose distribution for the actual fluence was calculated by using AAA dose calculation algorithm that has been shown to be superior against other algorithms.^(^ ^27^ ^)^ The final dose distributions were calculated with a grid size of $2.5\times 2.5\;{\text{mm}}^{2}$ with inhomogeneity correction. The dosimetric data for the nine IMRT plans were compared and analyzed for both energies for ten patients.

### B. Generalization of dose variations (HU=−1000, 0, and +1000)

The trend of dose variation as a function of HU change was studied by comparing individually optimized plans for the three different HU for ten prostate patients. The ten patients are selected to cover a wide range of anterio–posterior and left–right separations, as well as rectal and bladder volumes. Table 1 summarizes the physical measures and the volumes of interest for the ten patients.

## III. RESULTS

### A. Detailed study of effects of rectal balloon filling on dose distributions

**Figure 1:**
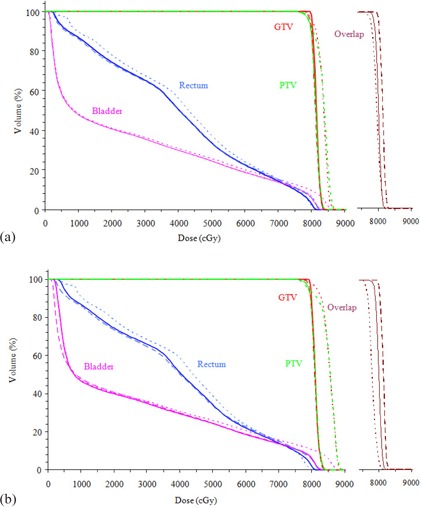
Dose‐volume histogram of GTV, PTV, rectum, and bladder for an air‐filled balloon (dotted line), a water‐filled balloon (solid line), and a contrast‐filled balloon (dashed line) for (a) 6 MV and (b) 15 MV X‐rays. For clarity, the DVHs of the overlap region are plotted separately in the panel on the right.

Figure 1(a) compares the DVH of the GTV, PTV and OARs for 6 MV X‐rays for the air‐filled, water‐filled, and contrast‐filled balloons. The largest difference is in the coverage of the GTV and PTV. The shoulders in the ${\text{GTV}}_{\text{air}}$ and ${\text{PTV}}_{\text{air}}$ DVHs indicate existence of underdose regions in the respective volumes with the air‐filled balloon. On the other hand, a larger hot spot also exists for the respective ${\text{GTV}}_{\text{air}}$ and ${\text{PTV}}_{\text{air}}$ for the air‐filled balloon compared to those corresponding to water‐ and contrast‐filled balloons. A similar difference of GTV and PTV between air‐filled and water‐filled balloon can be observed for 15 MV, though to a smaller magnitude (Fig. 1(b)).

**Figure 2:**
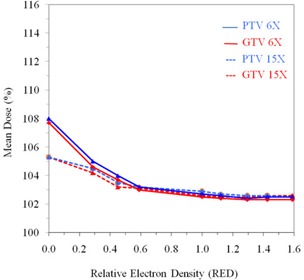
Variation in mean dose for GTV and PTV with relative changes in RED of rectal balloon for 6 MV and 15 MV.

The variation of the GTV and PTV coverage relative to change of RED can be clearly seen in Fig. 2, which shows the variation of the mean doses to ${\text{GTV}}_{\text{mean}}$ and ${\text{PTV}}_{\text{mean}}$ versus RED for 6 MV and 15 MV, respectively. The ${\text{GTV}}_{\text{mean}}$ and ${\text{PTV}}_{\text{mean}}$ are 107.0% and 108.0%, respectively, with an air‐filled rectal balloon. The mean dose falls rapidly as RED increases. With a water‐filled balloon, ${\text{GTV}}_{\text{mean}}$ and ${\text{PTV}}_{\text{mean}}$ doses are 102.5% and 102.7%, respectively, for the 6 MV photon beam (Fig. 2). There is minimal change in the mean doses of the target volume for RED higher than 1.0.

Figure 2 also shows the plot of the ${\text{GTV}}_{\text{mean}}$ and ${\text{PTV}}_{\text{mean}}$ versus RED for 15 MV, represented by dotted lines. The mean dose of PTV and GTV are both 105.3% with the air‐filled rectal balloon compared to 102.9% and 102.6%, respectively, with the water‐filled balloon. Again, there is very little improvement in the mean doses of the target volume with RED greater than one.

**Figure 3:**
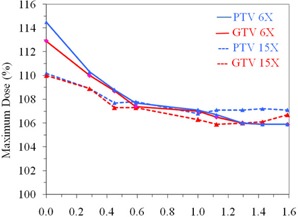
Variation of the GTV and PTV maximum dose with relative change in RED of rectal balloon for 6 MV and 15 MV.

A highly inhomogeneous plan with an air‐filled rectal balloon for 6 MV X‐rays produced a global max of 112.9% and 114.5% of the prescribed dose to ${\text{GTV}}_{\max}$ and ${\text{PTV}}_{\max}$, respectively. For the water‐filled balloon, the corresponding doses are 107.1% and 107.0%, respectively, as shown in Fig. 3. Figure 3 also shows an inhomogeneous plan for 15 MV (represented by dotted lines) with the air‐filled rectal balloon, producing a global maximum of 110.2% and 110.0% of the prescribed dose to ${\text{GTV}}_{\max}$ and ${\text{PTV}}_{\max}$, respectively, while the corresponding doses for the water‐filled balloon are 106.3% and 106.8%, respectively.

**Figure 4:**
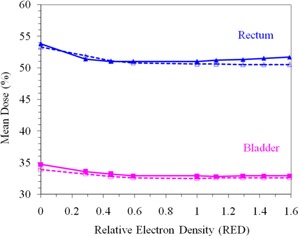
Variation of the rectum and bladder mean doses with relative change in RED of rectal balloon for 6 MV (solid line) and 15 MV (dotted line).

Figure 4 compares the variation of mean rectal and bladder dose with RED for both energies. The mean rectal dose changes by 2%−3% as the RED increases from 0 to 1.6 for both energies. This small change in the mean rectal dose is a result of the dose constraint to the rectum. On the other hand, variations of RED in the balloon from RED=0 MS to RED=1.6 have little effect on the bladder mean dose (Fig. 4). This is probably due to the large volume of the bladder.

**Figure 5:**
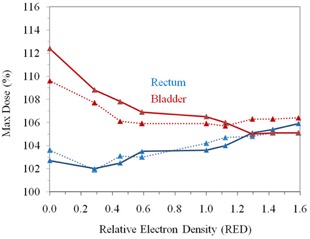
Variation of the rectum and bladder maximum dose with relative change in RED of rectal balloon for 6 MV (solid line) and 15 MV (dotted line).

Figure 5 shows that the maximum rectal dose increases with increasing RED, and is higher for 6 MV compared to 15 MV, which varies from 102.7% for RED=0 to 105.9% for RED=1.6. The location of the maximum dose is found to be in the anterior of the rectal balloon and in the target volume around the CAX for both energies, regardless of the rectal balloon filling. However, the maximum bladder dose ($B_{\max}$) decreases from 112% to 105% as the RED increases from 0 to 1.6 for 6 MV beam (Fig. 5). The larger variation of $B_{\max}$ may be attributed to a larger contribution from the anterior fields with the air‐filled balloon. As RED increases, the relative contribution from all fields slowly changes to become more evenly distributed as a result of dose optimization. The $B_{\max}$ variation is smaller for 15 MV beam, from 109.6% for RED=0 to 106.4% for RED=1.6.

**Figure 6:**
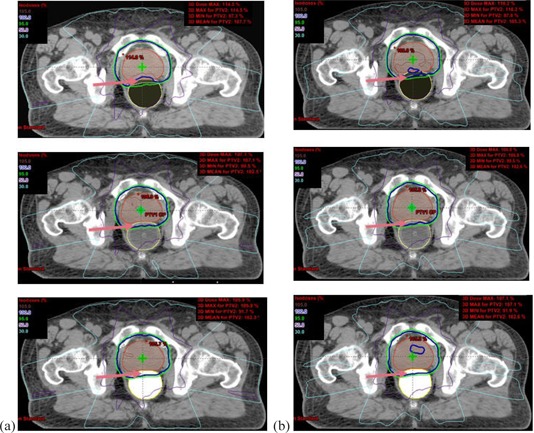
Comparison of central axis plane isodose distribution between an air‐filled (top), a water‐filled (middle), and a contrast‐filled (bottom) rectal balloon for (a) 6 MV and (b) 15 MV X‐rays. The arrow shows the structure, O, region of intersection between PTV and rectum.

Figures 6(a) and (b) show the central axis plane dose distributions for the air‐filled balloon, the water‐filled balloon, and the contrast‐filled balloon (RED=1.6) for 6 MV and 15 MV X‐rays, respectively. The arrow shows the structure, O, the region of intersection between PTV and rectum.

### B. Generalization of dose variations for HU=−1000, 0, and +1000

Table 2 summarizes the minimum, maximum, and mean doses in the overlap region for both 6 and 15 MV X‐rays for the ten patients selected for HU=−1000, 0, and 1000, respectively. Although the overlap region is not used in the optimization, its dose variation is directly impacted by the HU change in the rectal balloon filling.

**Table 2:** Summary of the minimum, the maximum, and the mean doses in the overlap region for both 6 MV and 15 MV X‐rays for the ten prostate patients.

	*Overlap Min.*			*Overlap Max.*			*Overlap Mean*		
*Patient*	HU=−1000	HU=0	HU=1000	HU=−1000	HU=0	HU=1000	HU=−1000	HU=0	HU=1000
6 MV									
1	88.9	93.4	95.1	102.7	103.6	105.9	98.5	101.0	102.8
2	95.9	98.8	99.2	102.9	104.7	105.7	99.5	101.3	102.1
3	93.7	97.3	96.1	100.6	103.9	103.6	97.7	100.9	101.0
4	95.4	98.6	98.6	101.3	104.8	104.3	98.0	101.4	101.3
5	94.9	99.5	100.2	101.2	103.7	105.2	99.1	101.8	102.2
6	94.9	100.0	99.4	101.3	103.4	104.6	98.4	101.6	101.9
7	94.8	97.5	97.7	101.0	104.5	106.6	98.7	101.6	102.0
8	93.9	99.4	99.2	102.5	105.1	103.9	98.4	101.5	101.7
9	92.2	98.9	100.0	99.0	101.6	104.2	96.0	100.6	102.5
10	93.6	98.7	100.1	102.0	102.0	103.8	97.0	101.0	102.2
Average	93.8	98.2	98.6	101.5	103.7	104.8	98.1	101.3	102.0
St. Dev.	2.0	1.9	1.7	1.2	1.2	1.0	1.0	0.4	0.5
15 MV									
1	90.6	92.6	96.3	103.6	103.6	105.1	100.4	100.4	102.7
2	97.6	98.7	99.2	102.8	103.3	105.7	100.6	101.0	102.1
3	93.9	95.8	97.1	101.9	102.9	103.5	99.7	100.9	101.1
4	97.2	99.3	99.8	102.6	103.7	103.3	100.9	102.3	102.0
5	97.4	99.3	99.4	102.7	104.1	103.9	100.5	101.9	101.8
6	96.2	98.6	99.5	101.8	103.1	104.0	100.1	101.5	101.9
7	96.1	98.2	98.1	103.2	102.5	103.9	100.3	100.6	101.0
8	97.6	99.5	99.8	104.0	103.7	103.8	100.3	101.5	101.8
9	96.8	99.5	99.2	101.8	103.2	104.2	98.8	101.8	101.4
10	96.3	98.3	100.1	102.4	102.1	103.6	100.0	101.2	102.9
Average	96.0	98.0	98.9	102.7	103.2	104.1	100.2	101.3	101.9
St. Dev.	2.2	2.2	1.3	0.8	0.6	0.7	0.6	0.6	0.6

For 6 MV X‐rays, the dose minimum varies from 88.9%−95.9%, with an average of93.8%±2% (1 SD) for an air‐filled balloon. With a water‐filled balloon, the minimum dose varies from 93.4%−100%, with an average of 98.2%±1.9% (1 SD). For the contrast‐filled balloon (HU=1000), the minimum dose varies from 95.1%−100.2% (1 SD) with an average of 98.6%±1.7%. Thus, while cold spot exists in the overlap region (and hence the PTV) for almost all ten patients over the full range of HU for the rectal balloon filling, an air‐filled balloon results in at least 5% colder dose regions in the PTV.

On the other hand, the maximum dose in the overlap region ranges from 99%−102.9%, with an average of 101.5%±1.2% for an air‐filled balloon. For a water‐filled balloon, the range of the maximum dose is 101.6%−105.1%, with an average of 103.7%±1.2%. For a contrast‐filled balloon (HU+1000), the variation of the maximum dose ranges from 103.6%−106.6%, with an average of 104.8%±1.0%. Thus, the maximum dose in the overlap region for an air‐filled balloon is generally 1%–5% lower compared to that of a water‐filled or a contrast‐filled (HU=1000) balloon.

The relatively lower minimum and maximum doses for an air‐filled balloon indicates that the mean dose in the overlap region is also lower than that for the water‐filled and the contrast‐filled balloons. Indeed, the mean dose for an air‐filled balloon varies between 96.0%−99.5%, with an average of 98.1%±1.0%. For a water‐filled balloon, the mean dose varies from 100.6%−101.8%, with an average mean dose 101.3%±0.4%; for a contrast‐filled balloon, the range is 101.0%−102.8%, with an average mean dose of 102% ±0.5%.

For 15 MV X‐rays, a similar variation for the minimum dose in the overlap region is observed. For a given patient, it is about 1%–5% lower for an air‐filed balloon compared to that of a water‐filled or a contrast‐filled balloon. However, the difference of the maximum dose is less than 3% for all ten patients over the full range of HU considered, and likewise for the mean dose.

Table 3 compares the variation of the mean dose to the rectum and the bladder for the ten patients for the three HUs: −1000, 0, and +1000. Also included are the mean PTV doses for the different patients. For both 6 and 15 MV X‐rays, the mean rectal and bladder doses averaged over the patients are practically the same (within 1 SD) regardless of the filling material in the rectal balloon. On the other hand, the average mean dose of the PTV is about 3% higher for the air‐filled balloon than that of the water‐filled and contrast‐filled balloons, whereas it is practically the same between the water‐filled and the contrast filled balloons.

**Table 3:** Summary of the mean doses in rectum, bladder, and PTV for the ten patients for both 6 MV and 15 MV X‐rays.

	*Rectum Mean*			*Bladder Mean*			*PTV Mean*		
*Patient*	HU=−1000	HU=0	HU=1000	HU=−1000	HU=0	HU=1000	HU=−1000	HU=0	HU=1000
6 MV									
1.0	53.8	51.0	51.7	34.7	32.9	32.9	107.7	102.5	102.3
2.0	62.6	60.8	61.1	18.2	17.5	17.6	105.5	102.0	102.3
3.0	61.0	60.0	59.6	58.4	57.8	57.8	103.9	102.0	102.0
4.0	62.2	61.6	61.5	74.1	73.5	73.8	104.2	102.6	102.3
5.0	49.8	48.4	48.7	50.9	49.1	49.5	104.7	101.9	102.4
6.0	50.8	50.4	50.6	19.0	18.9	18.5	105.3	102.2	102.2
7.0	48.5	47.1	47.2	47.0	45.5	45.7	104.3	102.0	102.5
8.0	48.7	47.6	47.8	40.1	39.1	39.4	104.9	102.5	102.7
9.0	53.6	55.1	55.1	30.2	29.5	28.9	105.5	102.0	101.8
10.0	46.2	45.5	46.7	47.1	44.3	44.3	106.9	101.5	101.8
Average	53.7	52.8	53.0	42.0	40.8	40.8	105.3	102.1	102.2
St. Dev.	6.1	6.1	5.9	17.4	17.2	17.4	1.2	0.3	0.3
15 MV									
1.0	53.3	50.6	50.5	33.9	32.5	32.6	105.3	102.6	102.6
2.0	62.1	59.8	59.9	17.2	16.7	16.8	104.1	102.2	102.4
3.0	60.7	59.1	58.9	58.2	57.7	57.9	103.4	101.9	101.9
4.0	62.6	61.1	60.6	73.3	72.8	72.9	103.4	102.5	102.5
5.0	50.3	48.9	48.5	51.1	50.4	50.4	104.1	102.6	102.6
6.0	50.5	48.2	48.0	18.4	17.8	17.9	104.2	101.9	101.9
7.0	48.4	45.9	46.0	46.8	44.8	45.1	104.6	102.1	102.2
8.0	49.7	48.0	48.0	40.3	39.5	39.6	104.0	102.4	102.5
9.0	56.3	55.3	54.2	30.0	29.2	28.8	106.0	101.5	102.4
10.0	46.5	45.2	45.8	46.2	44.5	44.6	105.4	102.2	102.2
Average	54.0	52.2	52.0	41.5	40.6	40.7	104.5	102.2	102.3
St. Dev.	6.0	6.1	5.9	17.5	17.5	17.5	0.9	0.4	0.3

## IV. DISCUSSION

The use of image guidance has significantly improved the accuracy in prostate irradiation by correcting the displacement of isocenter due to organ motion. However, the problem with prostate volume change as a result of varying rectal filling (to a lesser extent, the bladder filling) cannot be corrected by simply adjusting the positioning of the patient. Rectal balloon helps to stabilize the rectum and maintains a constant shape and volume of the rectum inside the treatment fields. Air‐filled balloon has been most commonly used in external beam prostate irradiation, while a water‐filled balloon has been used in proton therapy of the prostate.

In this study, we have examined the effects of the rectal balloon filling in detail on the coverage of GTV, PTV, and the doses to the OARs for a typical prostate patient treated with seven‐field IMRT plans. We have also carried out a systematic investigation on the effects of various rectal balloon fillings for ten prostate patients in order to understand the trend of dose variation due to the various rectal balloon fillings.

Analysis of the minimum, maximum, and mean doses for the PTV, GTV, and OARs for the different rectal balloon fillings (full range of HU/RED changes in the study) for both photon energies for a typical prostate patient (#1 in Table 1) shows a large dosimetric variation on GTV and PTV for RED change from 0 to 1.6 compared to that on bladder and rectum for both energies. The effect is larger for 6 MV X‐rays, in the range 3%–7% for the minimum, maximum, or mean doses as compared to 15 MV photons, which is around 2.5%−3.7%. The dosimetric change is also more pronounced in the range RED=0 to RED=0.45 compared to the changes above RED=0.45. Above RED=1, the change in dosimetry is around 1% or less. A contrast‐filled rectal balloon generally produces a more homogeneous target dose for both 6 MV and 15 MV X‐rays compared to those with a water‐filled balloon, but at the expense of a higher rectal dose and dose to the overlap region. The increase in mean dose to the overlap volume with increasing RED indicates an improvement in the PTV coverage as the RED of the balloon filling increases, as illustrated in Figs. 6(a) and (b).

It is also noticed that 3%–5% higher mean doses in rectum and bladder occurred with an air‐filled balloon for both energies compared to water‐filled and contrast‐filled balloons. Indeed, even with air‐filled balloon, hot spots of 102% and higher exist in the rectum laterally to the overlap region for both energies. These are probably due to dose optimization in IMRT trying to cover the posterior aspect of the PTV and GTV from the posterior oblique fields.

The higher maximum dose anteriorly and the larger underdosage region posteriorly as a result of the lack of scatter contribution from the air‐filled balloon also forces more doses to be delivered from the anterior fields (and to a lesser extent the posterior oblique fields, as described in the previous paragraph) in order to maintain the coverage within the optimization constraints.

When a similar study is carried out for a group of ten prostate patients, the magnitudes of the minimum, maximum, and mean dose are reduced, but nonetheless the dosimetric variation follows a similar trend as that for the detailed study for patient #1 over the range of HU used for the rectal balloon fillings. Despite the fact that there is no dose constraint on the overlap region between prostate and the anterior rectal wall, an air‐filled balloon results in a significant underdosage in the target volumes near the rectum–prostate interface, as compared to that of a water‐filled or contrast‐filled balloon for both 6 MV and 15 MV IMRT plans. An air–filled balloon results in 2% higher mean dose in the PTV for 6 MV and about 3% for 15 MV X‐rays compared to both water‐filled and contrast balloons. The rectum and the bladder mean doses are slightly higher (~1%–2%) with an air‐filled balloon compared to that for the water‐filled and contrast‐filled balloons for both 6 MV and 15 MV X‐rays. The differences in 6 MV and 15 MV plans are very small in general, which is in agreement with the data provided by Pirzkall et al.^(^ ^28^ ^)^

It is known that the AAA algorithm has a limitation in dealing with inhomogeneities and may result in 3% difference compared to measurements.^(^ ^27^ ^)^ This could be slightly improved with more advance algorithm like Acuros XB.^(^ ^29^ ^)^ However, since the entire calculations for all ten patients were carried out under the same condition for the same algorithm, the limitation of AAA in dealing with inhomogeneities has no effect on the comparison.

## V. CONCLUSIONS

An air‐filled balloon in general produces a higher underdosage in the PTV and the overlap region compared to that with a water‐filled or a contrast‐filled balloon. On the other hand, a contrast‐filled balloon produces higher minimum and maximum doses in the overlap region compared to those of a water‐filled balloon. The HU change in the rectal balloon filling, however, appears to have little effect on the doses to the OARs for both 6 and 15 MV X‐rays. Dosimetrically, a water‐filled or a contrast‐filled balloon is preferred over an air‐filled balloon.
